# Sexual behavior and awareness of Chinese university students in transition with implied risk of sexually transmitted diseases and HIV infection: A cross-sectional study

**DOI:** 10.1186/1471-2458-6-232

**Published:** 2006-09-18

**Authors:** Qiaoqin Ma, Masako Ono-Kihara, Liming Cong, Guozhang Xu, Saman Zamani, Shahrzad Mortazavi Ravari, Masahiro Kihara

**Affiliations:** 1 Center for Disease Control and Prevention of Zhejiang Province, Hangzhou, China; 2 Department of Global Health and Socio-epidemiology, Kyoto University School of Public Health, Kyoto, Japan; 3 Center for Disease Control and Prevention of Ningbo Municipality, Ningbo, China

## Abstract

**Background:**

The vulnerability of young people to HIV and the recent emergence of the HIV epidemic in China have made it urgent to assess and update the HIV/STD risk profile of Chinese young people.

**Methods:**

A self-administered questionnaire survey with cross-sectional design was conducted among 22,493 undergraduate students in two universities in Ningbo, China. Bivariate trend analysis and multiple logistic regression analysis were used to compare sexual behaviors and awareness between grades.

**Results:**

Of respondents, 17.6% of males and 8.6% of females reported being sexually active. Condom was reported never/rarely used by 35% of sexually active students in both genders in the previous year. Pregnancy and induced abortion had each been experienced by about 10% of sexually active female students and the female partners of male students, and about 1.5% of sexually active students of both genders reported being diagnosed with an STD. Multivariate analysis revealed that students in lower grades, compared to those in higher grades, were more likely to have become sexually active before university, to have become aware of sex before high school, and to have been exposed to pornographic media before the age of 17 years, and for sexually active respondents of both genders, to have engaged in sex without using a condom.

**Conclusion:**

Sexual behaviors of Chinese university students are poorly protected and sexual behaviors and awareness may have been undergoing rapid change, becoming active earlier and more risky. If this trend continues, vulnerable sexual network will grow among them that allow more expansion of sexually transmitted diseases and HIV.

## Background

Incidence of STD has recently dramatically increased in China. According to the national system of STD surveillance, from 1990–1998, the incidence of the eight STDs increased 3.7 times [1], and in 2004 gonorrhea and syphilis incidence ranked 4^th ^and 5^th ^among 27 notifiable infectious diseases, respectively [2]. At the same time, since the first case of HIV was identified in 1985, reported HIV/AIDS cases have been rapidly increasing in China, especially since 1997. While the increase in the reported cases of HIV could partly reflects expanded surveillance with improved reporting system, it also mirrors escalation of the HIV epidemic in China as the prevalence of HIV infection has also increased among drug users from 1.95% to 6.48%, and among commercial sex workers from 0.02% to 0.93% between 1996 and 2004 [3]. A Joint Assessment by the Ministry of Health and the United Nations Theme Group on HIV/AIDS in China estimated that as of the end of 2003, 840,000 people were living with HIV/AIDS, with an estimated 0.07% prevalence of HIV infection among the general population [4]. The Chinese HIV epidemic began in many districts among high-risk populations such as injection drug users, sex workers [5], and men who have sex with men [6,7]. Recent evidence has indicated that HIV has started to spread into a broader population through heterosexual transmission [4,5].

Young people are especially vulnerable to HIV. Researchers estimate that in many developing countries, one half or more of all HIV infections occur among young people less than 25 years [8]; this includes China, where about 60% of HIV infections are estimated to occur in young people aged 15–29 [9]. Effective HIV prevention among youth, therefore, is the key to the future course of the HIV epidemic and understanding sexual attitudes and behaviors of youth in terms of HIV/STD risk is critical in this respect.

Some studies have examined the sexual behaviors of university/college students in China. A survey performed in Beijing in 1989 found that 13% of male students and 6% of female students had some sexual experience [10]. In 1992, a study in Shanghai indicated that 18.8% of male students and 16.8% of female students had engaged in premarital sex [11], and a 1997 study showed that 15% of male and 13% of female university students in Beijing had experienced premarital sex [12]. In 2000, a nationwide survey involving over 5000 participants revealed that 11.3% of university students were sexually active [13]. While these studies provided useful information about the sexuality of Chinese university/college students, they were more related to sexology than to HIV/STDs, so they did not assess the risk for HIV/STD infection. The recent emergence of the HIV epidemic in China has made it urgent for researchers to assess and update the behavioral HIV/STD risk profile of Chinese young people.

This study was conducted to obtain detailed profiles of sexual attitudes and behaviors and associated factors among university students. The study was conducted in one large city in Zhejiang Province. This area has had one of the highest rates of gonorrhea and syphilis among all Chinese provinces, and reported cases of HIV have dramatically increased during recent years [2,14], making the development of effective prevention programs for youth a priority.

## Methods

### Participants and settings

This study was conducted in two large state-owned universities in Ningbo Municipality, Zhejiang Province, China. Ningbo is a key trading port city on the east coast with a population of 5.5 million and is one of the most economically developed areas in China. The city contains only two universities, both of which participated in the study. Both universities cover a wide range of disciplines such as economics, law, management, education, literature, science, engineering, and medicine.

All undergraduate students from grades I to IV in both universities were recruited. Here, grade means an academic year of study. Of the possible 29,409 respondents, 22,940 (78.0%) actually responded, and 447 were eliminated from the analysis due to apparent invalid responses, resulting in a final response rate of 76.5% (n = 22,493).

### Data collection

The questionnaire was developed through a review of Chinese and international literature and then modified using the results of qualitative studies that included in-depth interviews for 11 students (3 males and 3 females from university A and 3 males and 2 females from university B) recruited by university counselors from major faculties of two universities and four focus group interviews (for each gender in each university) among 21 students in total conveniently recruited by university counselors from major faculties of two universities. The revised instrument was pilot-tested among a group of 50 students in one of the two universities randomly selected from varying grades and faculties and from both genders. The instrument reliability was then evaluated in 89 of 160 college students recruited from another city who could be matched between two tests with a one-week interval. More than 70% of the categorical variables were found to have Kappa statistics over 0.4 (all *P *< 0.05) and the coefficients of correlation for discrete variables varied from 0.63 to 0.93 (all *P *< 0.05). For the main survey, students were requested by university staffs and student leaders to come to class rooms at specified times other than lecture hours.

Field teams consisting of trained staff from the local Centers for Disease Control and Prevention and counselors from each university collected data from November-December, 2003.

### Ethical considerations

The institutional review processes of Zhejiang Province's Center for Disease Control and Prevention and the two universities involved in the study reviewed this study's research protocol, including instruments, and approved its use; the Ningbo Education Board also approved it. As a process of informed consent, all students were advised of the study's purpose, and invited to the study being told where and when the survey was to be conducted. They are at the same time explained that non-participation would cause no disadvantage to them, and that participants' privacy and confidentiality would be strictly protected as no personal identifiers were included in the questionnaire and data was to be presented only in an aggregated manner. All these policies were also printed in the front page of the questionnaire.

### Statistical methods

Statistical analyses were performed using Epi-Info (Version 6.0, CDC, Atlanta, GA), SPSS for Windows (Version 12.01; SPSS Inc., Chicago, IL) and SAS for Windows release 8.02 (SAS Institute Inc., Cary, NC, USA). Difference in prevalence across the grades was assessed using a Chi square test for linear trend in proportion without adjusting for multiple testing because the tests were done for exploratory purposes for the subsequent multivariate analyses. Variables that exhibited significant linear trends, being greater in lower- than higher-grade students, were further assessed using multiple logistic regression analysis to adjust possible confounding. Because of the very large sample size, effect sizes less than 5% between the grades in both genders were not considered for multivariate analysis. Adjusted odds ratios and 95% confidence intervals summarizing the association between the selected variables and grades were calculated for each grade category. Contrast statistics were further performed to test the linear trend of odds ratios. Student's *t*-test and Chi square test were used when appropriate. A *P*-value of less than 0.05 was considered statistically significant for these analyses.

## Results

### Socio-demographics

The 22,493 respondents were almost evenly distributed between genders and universities. Of the students, 70% were aged 20 to 23 years with a mean age of 20.3 [standard deviation (SD) 1.4] (Table 1). Hometowns were roughly equally distributed between rural, towns, and urban areas. The majority of students was living in university dormitories and self-reported as coming from economically mid-level families.

### Sexual behaviors

Of all respondents, 17.6% of males and 8.6% of females were sexually active (Table 2) and the proportion of male students who had experienced sex before university was significantly greater in lower- than higher-grade students. The same trend, although involving a smaller proportion, was evident among female students. The mean age of first sexual experience was 19.4 (SD = 1.8) and 19.7 (SD = 1.6) for males and females, respectively (t = -3.37, P = 0.001).

Of sexually active respondents, 71.5% of males and 62.2% of females reported having sex in the previous year. Among those who had been sexually active in the previous year, males were significantly more likely to have had non-regular partners, including casual partners and commercial partners (x^2 ^= 40.80, *P *< 0.001) than were females; non-regular partnership was more common for lower than higher-grade students in both gender. Males were also significantly more likely to have had multiple partners than were females (x^2 ^= 10.21, *P *= 0.001); multiple partners per year were more common for lower- than higher-grade male students, but not for female students (Table 2). Condom was never/rarely used by 35% of sexually active students in both genders. Among male students, proportions of those who never or rarely used condom declined from first graders to forth grade students (*P *value for trend analysis < 0.001). However, no significant trend in condom use was found between high and low grade female students (P value for trend analysis = 0.08). About 57% of male and 49% of female students never or rarely used contraceptive pills in the previous year.

Regarding the gender of partners, over 85% of respondents reported only having sex with members of the opposite sex during their lifetime, 3.4% of male and 2.9% of female respondents reported having had homosexual and/or bisexual relationships, with the rest failed to report a clear answer. Pregnancy had been experienced by 9.9% of female students and by 9.8% of male students' female partners over their lifetime; the numbers for induced abortion were almost exactly the same in proportion as pregnancy, among students of both genders. The prevalence of sexually active students during lifetime diagnosed with an STD was around 1.5% for both genders.

### Awareness of sex, sexual attitudes, and exposure to pornographic media

Awareness of sex, sexual attitudes, exposure to pornographic media, and using Internet to meet a girl/boy friend are compared between the grades in both genders in Table 3. Students of both genders in lower grades reported becoming aware of sex earlier than did students in higher grades, where males who became aware of sex before high school declined from 65.6% in the first grade to 52.5% in the fourth grade students. The corresponding figures for females were 42.3% in the first grade and 26.7% in the fourth grade. The proportion of students who were exposed to each form of pornographic media (books/magazines/videos, and websites) was much greater in males than females and the proportion of male students who were exposed to each form of pornographic media before the age of 17 was significantly greater in lower- than higher-grade students. The Internet was also actively used to find dates; 51.1% of all male respondents and 43.8% of all female respondents reported having found a girl/boy friend over the Internet.

Up to 62% of males and 86% of females disapproved high school students having sex, with higher grade students disapproved it more than lower grade ones. Premarital sex was considered unacceptable by 12.1% of males and 30.8% of females, while commercial sex by 67.3% of males and 84.9% of females.

### Multiple logistic regression analysis

Multiple logistic regression analyses were performed for variables that showed a significantly greater trend for students in lower grades (exception for disapproval of sex at high school); these were used to assess possible confounding by university, faculty, residential area, and reported family economic status with fourth grade as a reference (Table 4). These analyses confirmed the results of bivariate analyses, showing that lower-grade students are likely to be more sexually active or unprotected, to have become aware of sex earlier, and to have been exposed to pornographic media earlier than the higher-grade students in both genders except for multiple partnerships among female students. Most striking differences between grades (odds ratio > 5) were observed in "having sex before university" among female students and "being exposed to pornographic website ≤ 16 year-old" among male students.

Type of sexual partner, multiple partnerships and condom use in the previous year were further analyzed adding the age of first sexual experience as an independent variable to assess possible confounding of the different proportions of early or late initiations into sex across the grades. This is because students who became sexually active early (before university) are more likely to ever have multiple partnerships and to ever have non-regular partners than were students who were initiated late (mean numbers of sexual partners for males in the previous year were 1.5 ± 1.1 [SD] and 1.2 ± 0.6 [SD] for early initiation and late initiation, respectively (*P *< 0.001), and 1.4 ± 0. 8 and 1.1 ± 0.4, respectively, among females (*P *< 0.001); proportions of having non-regular sexual partners for males in the previous year were 20.8% and 10.8% for early initiation and late initiation, respectively (*P *< 0.001), and 9.4% and 2.4%, respectively, among female (*P *< 0.001). These analyses showed that significant trend between the grade disappeared after the adjustment with the age of first sex in the type of sexual partner (odds ratios after further adjustment ranged between 0.84–1.03 for males and 0.87–1.27 for females) and in the number of sexual partners (0.73–1.03 for males) but not in the condom use in the previous year (1.0–1.73 for males and 1.0–1.98 for females, both *P *≤ 0.02). These results suggest that the trend of lower-grade students to have casual and/or commercial sexual partners and multiple partnerships more than higher-grade students are likely due to an increase in the number of students who initiate sex early, but the trend in unprotected sex is not.

## Discussion

Rates of sexual experience reported by the university students who responded to our study were 17.6% among males and 8.6% among females. These rates fall within the ranges reported by Chinese university/college students in other Chinese cities since 1995 [12,13,15]. During the last decade, the rates of sexual experience among Chinese university students do not appear to have undergone a dramatic change, remaining much lower than rates observed in the USA, Europe, and Japan during the 1990s and early 2000s [16-19]. This may be related to the fact that at the time of this survey, the Chinese Ministry of Education prohibited marriage among university students and the universities discouraged sex, although the Ministry's ban was effectively lifted in September 2005 [20]. However, our results clearly indicate that there have been changes in the sexual behaviors and awareness of university students, with both male and female students in lower grades becoming more aware of sex, having sex earlier, and having more casual/commercial and multiple partnerships. If this trend continues, it may expand the subpopulation of students who have multiple partners in a year, expanding the sexual network among them.

Our study also revealed prevalent unsafe sexual practices among respondents; 35% of respondents from both genders reported never/rarely-using condoms in the previous year. This, together with the low contraceptive pill use among responds, is probably the basis for the prevalence of both pregnancy and induced abortion, which were both as high as 10% among sexually active female respondents and the female partners of male respondents. Since pregnancy rates were almost identical to rates of induced abortion, it is possible that most pregnancies were artificially aborted, highlighting the importance of introducing safe sex education. In contrast to rates of induced abortion, the prevalence of sexually active students diagnosed with an STD during their lifetime was below 2% for students of both genders. This may be due to under-reporting, to the presence of certain STDs such as chlamydial and gonorrheal infections that remain largely asymptomatic especially among women [21,22], to embarrassment or financial costs preventing students from seeking medical care, to the limited availability of testing for chlamydial infections in the study area, or to the fact that the university students' sexual network was not developed enough to allow the spread of STDs.

Another important finding was that the condom use progressively lessened in lower-grade students of both genders. Though data are not shown in this paper, the same trends were significant for condom use over students' lifetimes as well as during their last sexual encounter. Since multiple logistic regression analyses revealed that these trends were all independent of the age of first sexual experience, it appears that sexually active younger university students are generally less protected than the older students. If this trend continues together with the increase in early sex initiation associated with more non-regular and more multiple partnership, the vulnerable subpopulation of students engaging in unsafe sexual practices will expand, potentially leading to an increased incidence of induced abortion and probably increased future STDs and HIV infection. This concern is supported by many previous studies indicating that an early age of sexual debut is associated with negative outcomes such as unwanted pregnancy, induced abortion, and STDs [23-25]. Research done in African countries has demonstrated that having sex at an early age is significantly associated with an increased incidence of HIV infection [26,27].

These trends of lower grade students to be sexually active earlier, to be aware of sex earlier, and to more accept adolescent sex compared to higher grade students were closely associated with the proportion of students who were exposed at a young age to pornographic media such as books/magazines/videos, and websites, suggesting that pornographic media may have had some influence on respondents' sexual awareness and practices. The rates reported having been exposed to pornography are similar to rates reported recently among young men in Hong Kong [28] and much higher than the prevalence (27.6%) reported among young Chinese adults in 1993 [29], implying that young Chinese people have become increasingly exposed to pornographic media over the last decade.

The Internet is a new, fast-developing media in China, and the population of users under 35 years of age has dramatically increased during the last several years (1.9 million in 1999 to 65 million in 2004 nationwide) [30]. This may be the cause of greater amounts of and earlier exposure to pornographic websites in lower-grade students. Our study also revealed that about half of our respondents in both genders had used the Internet to meet a girl/boy friend. Previous reports have shown that the use of the Internet for partner seeking and exposure to pornographic media are associated with risky sexual behaviors that lead to STD/HIV infection [28,31-33]. Careful monitoring will be needed in China regarding the possible future impact of the Internet and pornographic media on the sexual attitudes and behaviors of young people.

This study found striking gender differences (male > female) in rates of sexual experience, attitudes about premarital sex, and exposure to pornographic media; these differences were consistent with previous studies done in China [13,15,34-37], suggesting the importance of targeting prevention efforts toward male students. However, our study also suggested the need to carefully monitor possible changes among female students. Female students appear to be rapidly changing in sexual awareness and behavior, as the proportion of female students who became aware of sex before university was much greater in first- than in fourth-grade students, and the proportion of female first-grade students who reported having sex with non-regular partners in the previous year was over five-fold greater than that in fourth-grade students.

Finally, our study revealed that 3.4% of male students had experienced homosexual and/or bisexual activities. As well, 14.7% of males and 4.7% of females reported that their sexual encounters in the previous year involved commercial sex and casual sex. Though the proportions of these sexual practices were relatively small, prevention should clearly target these subpopulations since HIV/STDs epidemics has already been found in populations of men who have sex with men and in commercial sex workers in many parts of China [4-7]. The liberal attitudes both genders have about commercial sex and premarital sex are of serious concern in this respect, and should be adequately addressed in any future prevention program.

This study had several limitations. First, its cross-sectional design was limited in evaluating cause-and-effect associations. Second, the results obtained in this study should not be generalized to all Chinese young people or to all Chinese university students, since our sample was limited to university students within one municipality and socio-demographic or socio-economic characteristics are greatly diverse among Chinese provinces. Finally, the possible bias introduced by under-reporting should be noted, since missing data was disproportionately high (up to 20%) in questions related to sexual behaviors. A proportion of non-respondents may have considered questions about sexual behaviors to be too sensitive as all of them were unmarried.

## Conclusion

Our results suggests that, though sexual activity is still moderate among the respondents, sexual behaviors are poorly protected and sexual awareness and behaviors are under possible rapid changes becoming more active and risky, potentially driven by heavy exposures to various pornographic media and Internet from early age. Since the change was associated with earlier sex initiation, more commercial and casual sex, greater multiple partnerships and less condom use, vulnerable sexual network that allows the spread of STDs and HIV may grow among university students if these trend persist. Further surveys and surveillance on sexual behavior, its consequences, therefore, should be carried out among Chinese youth to develop targeted and effective prevention to protect them from adverse reproductive outcomes and STDs and future infection of HIV.

## Abbreviations

STD: Sexually Transmitted Disease

HIV: Human Immunodeficiency Virus

AIDS: Acquired Immune Deficiency Syndrome

## Competing interests

The author(s) declare that they have no competing interests.

## Authors' contributions

All authors contributed to the design of this research. MQ performed the statistical analysis and draft of the manuscript; CL and XG coordinated the study in field; ZS and SMR helped analyze the data; MOK and MK supervised the research, statistical analysis and revised the manuscript. All the authors of the manuscript have read and agreed to its content.

## Pre-publication history

The pre-publication history for this paper can be accessed here:



## Figures and Tables

**Table 1:** Socio-demographic characteristics of respondents by gender*

	**Male % (n = 11,255)**	**Female % (n = 11,238)**	**Total % (n = 22,493)**
**University**			
University A	50.8	53.6	52.2
University B	49.2	46.4	47.8

**Age (years)**			
16–19	23.6	33.8	28.7
20–23	74.6	65.5	70.0
≥24	1.1	0.4	0.8
Mean age ± SD†	20.5 ± 1.4	20.1 ± 1.3	20.3 ± 1.4

**Grade**			
Grade I	33.1	33.0	33.0
Grade II	29.4	30.0	29.7
Grade III	26.2	25.5	25.9
Grade IV	11.3	11.4	11.4

**Residence before university**			
Rural	32.3	24.2	28.3
Town	37.9	39.4	38.6
Urban	29.4	36.1	32.7

**Current residence**			
Dormitory	97.4	98.5	98.0
Other	2.1	1.2	1.7

**Perceived family's economic status**			
Rich	7.2	8.5	7.8
Intermediate	82.9	86.1	84.5
Poor	9.7	5.2	7.4

*The percentage of respondents may not add up to 100% due to non-response for some items.

†Standard deviation.

**Table 2:** Sexual behaviors of respondents by gender and grade

**Characteristics**	**Male (%)**						**Female (%)**					
	
	**Grade I**	**Grade II**	**Grade III**	**Grade IV**	**Total**	*P *value*	**Grade I**	**Grade II**	**Grade III**	**Grade IV**	**Total**	*P *value*
**Sexual experience†**												
Yes	11.7	16.7	23.8	22.6	17.6	<0.001	5.4	7.7	11.6	13.1	8.6	<0.001
No	88.3	83.3	76.2	77.4	82.4		94.6	92.3	88.4	86.9	91.4	

**Time of first sexual intercourse†**												
Before university	8.9	7.2	7.2	2.8	7.3	<0.001	2.9	1.7	1.7	0.3	1.9	<0.001
University	2.5	8.9	15.6	19.1	9.7		2.1	5.6	9.4	11.9	6.1	
Not specified	0.3	0.6	1.0	0.7	0.6		0.4	0.4	0.6	0.9	0.5	

**Partner type (in previous year)‡**												
Ever commercial/casual partner	18.8	16.3	13.1	10.2	14.7	0.002	9.2	5.0	3.8	1.7	4.7	0.007
Regular partner only	79.3	80.2	84.2	88.1	82.7		87.2	90.6	94.8	95.8	92.5	
Missing	1.9	3.5	2.7	1.8	2.6		3.7	4.4	1.4	2.5	2.8	

**Number of sexual partners (in previous year)‡**												
≥2	22.4	18.5	17.2	10.6	17.6	0.001	11.0	13.2	11.8	12.7	12.2	0.85
1	70.2	70.1	74.1	84.1	73.8		77.1	81.1	82.2	81.4	80.8	
Missing	7.4	11.4	8.8	5.3	8.6		11.9	5.7	6.1	5.9	7.0	

**Condom use (in previous year)‡**												
Never/Rarely	46.3	35.9	31.0	27.9	35.1	<0.001	43.1	35.8	31.9	33.1	35.2	0.08
Sometimes	23.9	15.8	19.9	15.9	19.1		14.7	16.4	19.7	20.3	18.0	
Often/Always	25.6	41.3	41.9	50.0	39.5		32.1	32.7	35.7	35.6	34.2	
Missing	4.2	7.1	7.2	6.2	6.4		10.1	15.1	12.7	11.0	12.5	

**Pill use (in previous year)‡**												
Never/Rarely	58.9	53.0	58.1	58.0	56.9	0.81	46.8	47.8	53.1	47.5	49.4	0.63
Sometimes	21.0	19.8	17.0	11.1	17.7		17.4	18.9	14.6	21.2	17.5	
Often/Always	17.5	23.9	21.2	28.3	22.2		30.3	29.6	28.6	28.0	29.0	
Missing	2.6	3.3	3.7	2.7	3.2		5.5	3.8	3.8	3.4	4.0	

**Gender of sexual partner (lifetime)¶**												
Homosexual/Bisexual	2.3	3.8	4.0	3.1	3.4	0.32	3.5	5.4	1.2	1.8	2.9	0.04
Heterosexual only	91.3	86.8	87.8	90.6	88.7		82.6	83.5	88.3	85.1	85.3	
Missing	6.4	9.4	8.3	6.3	7.9		13.9	11.2	10.5	13.1	11.8	

**History of pregnancy of partner or themselves (lifetime)¶**												
Yes	6.6	9.4	11.0	12.5	9.8	<0.01	8.5	8.8	11.4	10.1	9.9	0.40
No/Don't know	87.0	82.1	81.4	81.9	82.9		77.1	79.6	77.8	78.0	78.2	
Missing	6.4	8.5	7.7	5.6	7.3		14.4	11.5	10.8	11.9	11.9	

**History of induced abortion of partner or themselves (lifetime)¶**												
Yes	6.6	7.8	10.7	12.9	9.3	0.001	9.5	9.2	10.5	10.7	10.0	0.65
No/Don't know	86.5	82.8	81.8	81.5	83.1		75.6	79.6	78.4	77.4	78.0	
Missing	6.9	9.4	7.5	5.6	7.6		14.9	11.2	11.1	11.9	12.0	

**History of being diagnosed as having STDs (lifetime)¶**												
Yes	1.1	1.4	1.3	1.0	1.3	0.88	1.0	1.9	1.8	0.6	1.5	0.80
No	88.6	82.8	87.1	88.9	86.5		80.1	81.5	83.8	81.0	81.9	
Missing	10.3	15.7	11.7	10.1	12.3		18.9	16.5	14.4	18.5	16.6	

* Chi square test for linear trend in proportion.

† Among all respondents.

‡ Among respondents who had sex in the previous year (n = 309, 368, 513, and 226 for male grade I, II, III and IV students, respectively, and n = 109, 159, 213, and 118 for female grade I, II, III, and IV students, respectively).

¶ Among all sexually active respondents (n = 437, 553, 703, and 287 for male grade I, II, III, and IV students, respectively, and n = 201, 260, 334, and 168 for female grade I, II, III, and IV students, respectively).

**Table 3:** Period of first awareness of sex, exposure to pornographic media, attitude toward sex by gender and grade

**Characteristics**	**Male (%)**						**Female (%)**					
	
	**Grade I **(n = 3728)	**Grade II **(n = 3308)	**Grade III **(n = 2949)	**Grade IV **(n = 1270)	**Total **(n = 11255)	*P *value*	**Grade I **(n = 3708)	**Grade II **(n = 3375)	**Grade III **(n = 2869)	**Grade IV **(n = 1286)	**Total **(n = 11238)	*P *value*
**Period became aware of sex**												
Before high school	65.6	59.0	52.8	52.5	58.8	<0.001	42.3	32.3	30.0	26.7	34.4	<0.00
High school	32.6	36.6	39.3	37.2	36.1		51.9	52.3	45.1	41.4	49.1	1
University	1.3	3.8	7.4	9.8	4.6		5.5	15.1	24.7	31.4	16.2	
Missing	0.4	0.7	0.4	0.6	0.5		0.3	0.3	0.2	0.5	0.3	

**First exposure to pornographic media†**												
Early (≤ 16 year-old)	44.4	31.8	29.8	28.5	35.1	<0.001	10.0	6.8	5.6	3.4	7.2	<0.00
Late (≥ 17 year-old)	27.3	40.4	46.2	52.3	38.9		7.6	13.4	19.9	22.3	14.2	1
Never	19.1	11.2	8.4	8.3	12.8		80.4	77.5	71.0	70.9	76.0	
Age unknown/Missing‡	9.2	16.6	15.6	10.9	13.2		2.0	2.4	3.4	3.3	2.6	

**First exposure to pornographic website**												
Early (≤ 16 year-old)	13.7	7.0	3.2	1.8	7.6	<0.001	0.9	0.5	0.1	0.2	0.5	<0.00
Late (≥ 17 year-old)	42.5	55.0	67.1	75.4	56.3		5.0	8.2	15.4	15.9	9.8	1
Never	35.7	22.3	14.9	11.5	23.6		93.3	90.1	81.9	81.3	88.1	
Age unknown/Missing‡	8.1	15.7	14.9	11.3	12.5		0.9	1.2	2.5	2.6	1.6	

**Meet a girl/boy friend over the Internet**												
Yes	51.3	49.4	52.2	52.2	51.1	0.31	42.9	40.6	47.1	47.0	43.8	<0.00
No	48.4	50.3	47.5	47.6	48.6		56.9	59.3	52.9	52.9	56.1	1
Missing	0.3	0.3	0.3	0.2	0.3		0.1	0.1	0.1	0.1	0.1	

**Approve of sex during high school**												
Disapprove	61.1	60.9	61.5	71.1	62.3	<0.001	84.0	87.3	86.8	88.5	86.2	<0.00
Approve/Don't know	37.2	35.4	35.6	27.6	35.2		15.4	12.0	12.3	10.3	13.0	1
Missing	1.7	3.7	2.9	1.3	2.6		0.6	0.7	0.9	1.2	0.8	

**Approve of sex before marriage**												
Disapprove	14.1	12.0	9.4	12.4	12.1	<0.001	36.4	29.7	26.7	26.2	30.8	<0.00
Approve/Don't know	83.4	83.5	86.6	85.8	84.5		62.6	69.0	71.9	72.2	68.0	1
Missing	2.5	4.5	4.0	1.7	3.4		1.0	1.3	1.3	1.6	1.2	

**Approve of commercial sex**												
Disapprove	69.9	68.4	63.4	65.3	67.3	<0.001	86.5	85.4	83.2	83.1	84.9	<0.00
Approve/Don't know	28.1	27.8	33.4	32.9	29.9		12.9	13.9	15.8	15.6	14.2	1
Missing	2.0	3.8	3.2	1.8	2.8		0.7	0.7	1.0	1.2	0.8	

*Chi square test for linear trend in proportion.

† Including pornographic novel, magazine, and/or video.

‡ Missing cases are less than 1% for each grade among both male and female students.

**Table 4:** Multivariate analysis on the association between risk characteristics and grade, divided by gender

**Risk characteristics**	**Male**					**Female**				
	
	**Grade I**	**Grade II**	**Grade III**	**Grade IV**	*P *value*	**Grade I**	**Grade II**	**Grade III**	**Grade IV**	*P *value*
				
	Odds ratios (95% CI)†					Odds ratios (95% CI)**†**				
**Having first sex before university **(referred to never or having first sex at university)	2.08 (1.43–3.02)	1.79 (1.23–2.59)	1.89 (1.31–2.75)	1	< 0.001	7.73 (2.80–21.33)	4.52 (1.63–12.58)	4.82 (1.73–13.44)	1	< 0.001
**Ever had sex with a casual and/or commercial sex partner in previous year**‡ (referred to having had only regular partner)	1.89 (1.03–3.47)	1.76 (1.01–3.10)	1.33 (0.78–2.29)	1	0.02	2.37 (0.40–14.04)	2.20 (0.42–11.68)	1.89 (0.36–9.92)	1	0.33
**Having had ≥2 sexual partners in previous year**‡ (referred to having had only one partner)	2.08 (1.16–3.72)	1.86 (1.08–3.20)	1.60 (0.96–2.68)	1	0.01	0.56 (0.22–1.42)	0.77 (0.36–1.68)	0.72 (0.34–1.50)	1	0.26
**Never or rarely used condom in previous year**‡ (referred to sometimes/often/always used)	1.87 (1.20–2.90)	1.39 (0.93–2.08)	1.14 (0.78–1.66)	1	0.003	1.98 (1.04–3.78)	1.43 (0.82–2.51)	1.12 (0.66–1.89)	1	0.03
**Became aware of sex before high school **(referred to becoming aware of sex in high school/at university)	1.66 (1.45–1.91)	1.27 (1.11–1.46)	0.98 (0.86–1.13)	1	< 0.001	2.11 (1.82–2.45)	1.37 (1.18–1.59)	1.19 (1.03–1.39)	1	< 0.001
**Being exposed to pornographic media ≤16 year-old**¶ (referred to never or being exposed ≥ 17 year-old)	1.66 (1.43–1.93)	1.12 (0.96–1.30)	1.02 (0.88–1.19)	1	< 0.001	3.24 (2.33–4.51)	2.09 (1.49–2.92)	1.69 (1.20–2.38)	1	< 0.001
**Being exposed to pornographic website ≤16 year-old **(referred to never or being exposed ≥ 17 year-old)	6.09 (3.94–9.41)	3.29 (2.12–5.13)	1.44 (0.90–2.30)	1	< 0.001	3.66 (1.07–12.58)	2.16 (0.61–7.58)	0.59 (0.13–2.66)	1	< 0.01
**Disapproved high school students' sex **(referred to approved or did not know)	0.93 (0.80–1.08)	0.91 (0.78–1.06)	0.84 (0.72–0.98)	1	0.09	0.86 (0.69–1.06)	1.06 (0.86–1.32)	0.96 (0.78–1.20)	1	0.27

* *P *value is calculated using linear contrast statistics.

† Odds ratio with 95% confidence interval in parentheses is adjusted for university, faculty, residential area, and family's economic status.

‡ Among students sexually active in the previous year.

¶ Including pornographic novel, magazine, and/or video.
